# Grade III bone cement implantation syndrome in malignant lung cancer patient: a case report

**DOI:** 10.1186/s12871-018-0492-x

**Published:** 2018-03-02

**Authors:** Pawan Kumar Hamal, Puspa Raj Poudel, Janith Singh

**Affiliations:** 1 National Academy of Medical Sciences, National Trauma Center, Kathmandu, Nepal; 2 National Academy of Medical Sciences, Bir Hospital, Kathmandu, Nepal

**Keywords:** Bone cement implantation syndrome, Carcinoma lung, Hip fracture

## Abstract

**Background:**

Bone cement implantation syndrome is a known complication causing mortality during perioperative period particularly in patients with malignancy. With rise in aging population with malignancy in low income country, the syndrome is more likely to be encountered.

**Case presentation:**

We present a case of 66 years old male patient with metastatic bronchogenic carcinoma of lung with pathological proximal femur fracture of left hip that underwent a cemented endoprosthesis under combined spinal epidural anesthesia who succumbed to intraoperative mortality due to grade III bone cement implantation syndrome even after aggressive fluid resuscitation, vasopressor use, and mechanical ventilation.

**Conclusions:**

Careful identification of risk factors with aggressive vigilance and intervention in part of surgeons and anesthesia both during intraoperative and postoperative period can mitigate the risk of bone cement implantation syndrome.

## Background

There was no agreed definition of bone cement implantation syndrome (BCIS) until it was proposed to be characterized by hypoxia, hypotension or both and/or unexpected loss of consciousness occurring around the time of cementation, prosthesis insertion, reduction of the joint or, occasionally, limb tourniquet deflation in a patient undergoing cemented bone surgery [1]. Three grades of syndrome have been proposed by Donaldson according to blood pressure measurement, degree of hypoxia and consciousness level corresponding to worse prognosis with final grade requiring cardiopulmonary resuscitation [1–3]. Those with advanced age, poor cardiopulmonary reserve, high ASA grade [3], pulmonary hypertension, bony metastasis, osteoporosis, pathological or intertrochanteric fractures, surgeries undergoing cemented prosthesis are implicated to be at increased risk (Table 1) [1, 2]. With increasing life expectancy, the burden of hip fractures is epidemiologically projected to increase. This worldwide annual number will rise to 6.26 million by the year 2050 [4]. In low income countries, aging population with comorbidities and rising burden of lung cancer [5] and practices of cemented arthroplasty, the syndrome complex is more likely to be encountered.

**Table 1:** Risk factors [1]

Preexisting disease	Surgical factors
Pre-existing pulmonary hypertension	Pathological fracture
Significant cardiac disease	Inter-trochanteric fracture
New York Heart Association class 3 or 4	Long-stem arthroplasty
Canadian Heart Association class 3 or 4	

## Case presentation

**Fig. 1:**
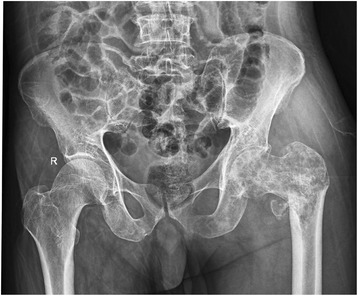
Anteroposterior view of X-ray pelvis suggesting multiple metastasis and pathological fracture of left hip

**Fig. 2:**
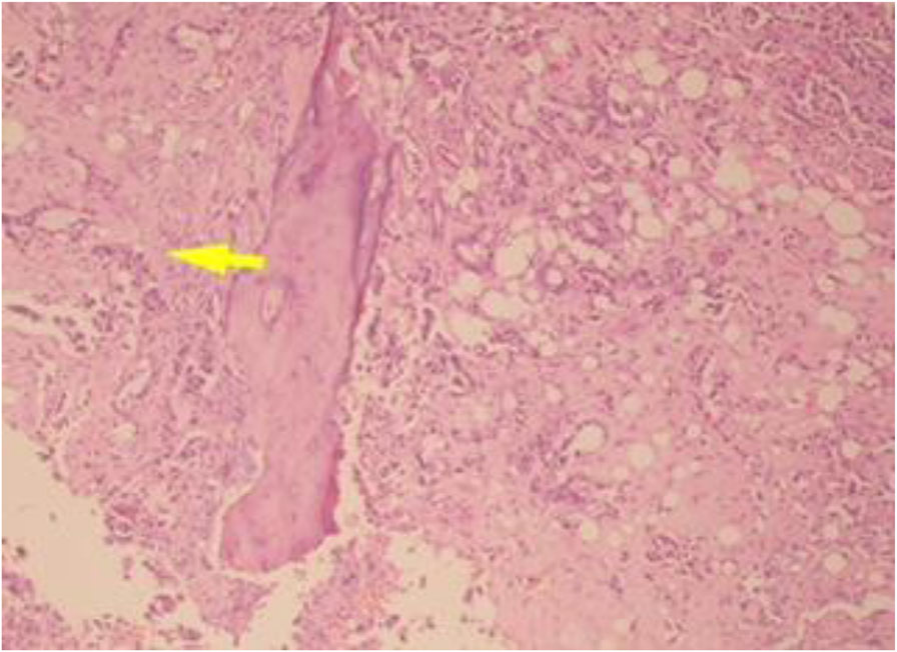
Hip biopsy show atypical cells arranged in glands, infiltrating stroma and entrapment of bony trabeculae

We present a case of 66 years old male from Nuwakot district of Nepal, farmer by occupation, referred from private hospital to our center with pain in left hip, on and off for last 5 months with suspected malignancy. The patient had normal X-ray findings. He also didn’t respond to analgesic regimen. Repeat X-ray of the pelvis (Fig. 1) shows pathological lesion suggestive of suspicious malignancy in neck of femur with differential of metastatic lymphoma or multiple myeloma. He also had history of pulmonary tuberculosis treated with chemotherapy 20 years back. He was a heavy smoker for last 30 years and occasionally takes alcohol. Initial workup for multiple myeloma including M-bands was negative. Biopsy of the hip suggested metastatic adenocarcinoma (Fig. 2). Immunohistochemistry of the specimen showed Bronchogenic origin. By this time, patient had difficulty bearing weight and was admitted for impending fracture of proximal femur with traction in situ.

On general examination patient’s vitals were within normal limits. He was pale. Airway examination was normal with Mallampati grade II. Systemic examination revealed normal cardiorespiratory findings except for some occasional crepitation at bases of both lungs with normal neurology and abdominal findings. Hematological parameters showed low hemoglobin of 9.3 g% with normal total count, differential counts, platelets, prothrombin time and international normalized ratio, activated Partial thromboplastin Time but had raised Erythrocyte Sedimentation Rate of 51 mm/h. His renal function test was normal. Chest X-ray showed healed Koch’s lesion on right upper lung field. Electrocardiogram (ECG) was within normal limits. Contrast computed tomography of chest, abdomen and pelvis showed calcified lesions on bilateral lung fields, multiple calcified hilar lymph nodes, paraaortic nodes, multiple lytic lesions at vertebral body at levels from T1-L5, bilateral Ilium, ischium, bilateral femoral heads, left proximal femur and pubis, with pathological fracture of proximal femur.

### Preoperative

Patient was evaluated as stage IV lung cancer with multiple metastasis according to AJCC (American Joint Committee on Cancer) 7th edition with almost 20% having 1 year survival [6]. Patient and his family members were counselled and they agreed for endoprosthesis with cemented Austin Moore prosthesis on palliative ground. They also consented for high risk of possible perioperative mortality during the procedure and due to bone cement implantation syndrome. Patient was categorized as American Society of Anesthesiologist grade III and planned under combined spinal epidural anesthesia with invasive arterial pressure monitoring.

### Intraoperative

I.V. access was taken with 16G cannula in left hand and 18G cannula in right hand. Baseline vitals were noted as right radial artery Invasive Blood Pressure (IBP): 125/70 mmHg, Heart rate (HR): 95 beats/min, Oxygen saturation (SPO_2_): 98% in room air and normothermic. Epidural catheter was kept at L3-L4 level using Loss of resistance technique with catheter tip at L1 with test dose confirmation with view of postoperative analgesia and prolonged surgery. Subarachnoid block was given with 26G Quincke’s needle at L4-L5 space with total 2.5 ml volume containing 0.5% heavy Bupivacaine (2.3 ml) and 100 micrograms of morphine (0.2 ml). The level of sensory block using pinprick method was recorded as T10 at 10 min. Patient was then kept at right lateral position. Hemodynamics were stable till the time of reaming, but just at the time of cementation, patient developed sudden onset bradycardia recorded HR: 30–35 beats/min, radial artery Blood Pressure of 50/30 mmHg and the patient was drowsy and unresponsive. I.V. Adrenalin was given in aliquots of 10 microgram with total 40 microgram and fluid bolus of 1 l with Normal Saline concomitantly. HR improved to 50–55/min, irregular, arterial blood pressure was still low and SPO_2_ was unrecordable. Endotracheal Intubation was done with Ketamine 100 mg and mechanically ventilated with 100% oxygen. By that time prosthesis was already inserted and joint reduced, with view of abandoning procedure quickly. Intravenous(I.V.) Hydrocortisone stat, I.V. Chlorpheniramine maleate, and I.V. Adrenalin 100 microgram (3 times) was given with view of possible anaphylaxis to bone cement. Arterial blood gas showed severe metabolic acidosis (PH 6.8, HCO_3_: 6, PCO_2_: 18 lactate: 10). Fluid resuscitation continued with Normal Saline, Adrenalin 20 mcg/min, Noradrenalin 20 mcg/min and I.V. Sodium Bicarbonate. HR and IBP was persistently decreased, SPO_2_ was undetectable and end tidal carbon dioxide (ETCO_2_) was in baseline with nonpalpable carotid but with ECG trace showing rhythm. Cardiopulmonary resuscitation was started with view of electromechanical dissociation for two cycles and patient returned with HR: 115/min, IBP: 80/50 mmHg and detectable ETCO_2_ and Oxygen Saturation and feeble carotid pulse. Central line was then placed in right femoral vein and I.V. Vasopressin was added in infusion. After 15 min, the patient deteriorated again and cardiopulmonary resuscitation (CPR) initiated. Patient revived after 2 cycles. Arterial Blood Gas (ABG) at this moment showed PH of 7.02, HCO_3_: 9, PCO_2_: 38, PaO_2_: 109, lactate: 9.5. Broad spectrum antibiotics was given and normothermia was maintained. After 30 min of hemodynamic instability patient again succumbed to asystole, but didn’t revive thereafter. He was later declared as dead with possible cause as bone cement implantation syndrome.

## Discussion and conclusions

Hip fractures pose a medical, societal, and economic burden with only one third gaining the functional recovery and one third succumbing to mortality [7]. Furthermore, those undergoing hemireplacement arthroplasty has incidence of syndrome as 20% [2] with cemented prosthesis showing 0.11% mortality risk according to registry reports occurring mostly at the time of cementation [1]. Mortality figures were even higher as 0.5% in Norway study [8]. The mortality risk for grade III BCIS as in our case was as high as 88% (Table 2) [2]. Most of the figures were based upon observational study with most data extracted from arterial blood pressure measurement, oxygen saturation data charted on anesthetic records [3]. Our observation was also based upon Invasive blood pressure measurement, oxygen saturation data, and clinical observation of mental status and taking into account the proposed classification by Donaldson [1]. Additionally, comorbid condition mainly cardiac, respiratory, malignancy, osteoporotic changes increase the risk of the syndrome complex [1, 2]. Literature review hypothesize the multifactorial model of pathogenesis according to postmortem finding of patient succumbing to intraoperative mortality for the syndrome complex which shows massive pulmonary fat embolism as a major culprit along with mast cell activation [9–11]. Anaphylaxis with release of mediators [12, 13] to monomer of bone cement, embolus [10] has also been proposed giving similar pictures as embolic model. There are also reports of patients requiring intensive care [14] and succumbing to death postoperatively [9, 15] particularly in patients who were unstable in intraoperative periods. It is difficult to come to conclusion of etiology in our case as we didn’t have the luxury of transesophageal echocardiography as well as the family members didn’t consent for postmortem examination but probably a multifactorial model would explain the phenomenon. Adverse clinical events with coma and death has been reported in patient having femoral metastasis who underwent cemented arthroplasty [16].

**Table 2:** Proposed grading [1] adverse events [21] and estimated 30-day mortality [2]

Grades of BCIS	Clinical findings (Donaldson)	Incidences of adverse events	Estimated 30-day mortality
Grade I	Moderate hypoxia (SPO_2_ < 94%) or Hypotension [fall in systolic blood pressure (SBP) > 20%]	~ 20%	9.3%
Grade II	Severe hypoxia (SpO_2_ < 88%) or Hypotension (fall in SBP > 40%) or Unexpected loss of consciousness.	~ 3%	35%
Grade III	Cardiovascular collapse requiring Cardiopulmonary Resuscitation	~ 1%	88%

Risk reduction can only be achieved with vigilant monitoring from both the surgeon and the anesthesiologist (Table 3) [3, 17]. Cemented arthroplasty although increases the mortality rate at first day, however has better mortality indices thereafter and improves pain and refractures rate compared to uncemented arthroplasty [18]. Careful reaming of the femoral canal with use of cement gun is recommended for frailer patient [19] which is less likely to happen in our set up. Better hemodynamic monitoring, early recognition and aggressive resuscitation and changes in surgical technique are recommended for prevention of catastrophic outcome [15]. Insertion of arterial pressure measurement, use of end tidal carbon dioxide particularly in vulnerable group gives more liberty to address the hemodynamic stability early [3]. If the patients develop the syndrome, cardiovascular collapse should be treated as Right ventricular failure with use of alpha-1 agonist and maintaining preload [1, 20].

**Table 3:** Three-stage process to reduce the incidence of problems in patients undergoing cemented hemiarthroplasty for proximal femoral fracture [17]

1. Identification of patients at high risk of cardiorespiratory compromise:	
a. Increasing age;	
b. Significant cardiopulmonary disease;	
c. Diuretics;	
d. Male sex.	
2. Preparation of team(s) and identification of roles in case of severe reaction:	
a. Pre-operative multidisciplinary discussion when appropriate;	
b. Pre-list briefing and World Health Organization Safe Surgery checklist ‘time-out’.	
3. Specific intra-operative roles:	
a. Surgeon:	
• Inform the anesthetist that you are about to insert cement;	
• Wash and dry the femoral canal;	
• Apply cement retrogradely using the cement gun with a suction catheter and intramedullary plug in the femoral shaft;	
• Avoid excessive pressurisation.	
b. Anesthetist:	
• Ensure adequate resuscitation pre- and intra-operatively;	
• Confirm to surgeon that you are aware that he/she is about to prepare/apply cement;	
• Maintain vigilance for signs of cardiorespiratory compromise. Use either an arterial line or non-invasive automated blood pressure monitoring set on the ‘stat’ mode during/shortly after application of cement;	
• Early warning of cardiovascular collapse may be heralded by a drop in systolic pressure. During general anesthetic, a sudden drop in end-tidal pCO_2_ may indicate right heart failure and/or catastrophic reduction in cardiac output;	
• Aim for a systolic blood pressure within 20% of pre-induction value;	
• Prepare vasopressors in case of cardiovascular collapse.	

Patient with femoral metastasis undergoing cemented arthroplasty are at high risk of developing high grade BCIS. Careful identification of risk factor with aggressive perioperative monitoring and changes in surgical technique can reduce the risk of bone cement implantation syndrome.

## Abbreviations

ABG: Arterial blood gas
AJCC: American Joint Committee on Cancer
BCIS: Bone cement implantation syndrome
CPR: Cardiopulmonary resuscitation
ECG: Electrocardiogram
ETCO_2_: End tidal carbondioxide
HR: Heart rate
I.V.: Intravenous
IBP: Invasive blood pressure
SBP: Systolic blood pressure
SPO_2_: Oxygen saturation
